# Hepatitis C Virus Genotype 4 in Ugandan Children and Their Mothers

**DOI:** 10.3201/eid1209.041068

**Published:** 2006-09

**Authors:** Robert J. Biggar, Betty A. Ortiz-Conde, Rachel K. Bagni, Paul M. Bakaki, Cheng-Dian Wang, Eric A. Engels, Sam M. Mbulaiteye, Christopher M. Ndugwa

**Affiliations:** * National Cancer Institute, Bethesda, Maryland, USA;; † National Cancer Institute–Frederick, Frederick, Maryland, USA;; ‡ Makerere University Medical School and Mulago Hospital, Kampala, Uganda

**Keywords:** hepatitis C virus, genotype 4, Africa, epidemiology, phylogeny, sequencing, sickle cell anemia, transmission

## Abstract

In Kampala, Uganda, in 2001, hepatitis C virus antibodies were found in 27 (4%) of 603 children and in 62 (12%) of 525 of their mothers. However, only ≈10% of positive results were confirmed by reverse transcription–PCR, which suggests frequent false-positive results or viral clearance. All sequenced types were genotype 4.

The prevalence of hepatitis C virus (HCV) infection in sub-Saharan African populations is an estimated 3%, with modest regional variation (*1*). The true prevalence may be lower because some positive HCV ELISA results may be due to cross-reactivity, and the reliability of recombinant immunoblot assay (RIBA) as a confirmatory assay is unclear (*2**,**3*). Few studies have confirmed antibody-positive results by molecular methods, and data on HCV genotypes in Africans are limited.

In Uganda, 2 studies showed a low HCV seroprevalence (*2**,**4*), but neither examined samples for viral RNA. In 2001, we conducted a survey of Ugandan children with sickle cell disease and their mothers and found that human herpesvirus 8 infection was associated with transfusion in these children (*5*). Children with sickle cell anemia receive frequent injections during their care, and transfusions are frequently required for anemia. We therefore assumed that our population would be at increased risk for HCV infection. We tested for HCV antibodies and, on antibody-positive samples, sought HCV RNA to confirm antibody reactivity. For RNA-positive samples, we identified genotypes by sequencing and phylogenetic analysis.

## The Study

In 2001, we enrolled 603 children (1–16 years of age) attending the Sickle Cell Clinic at Mulago Hospital, Kampala, in our study. By design, approximately half of the children had a history of blood transfusion. When available, the mothers of these children were also enrolled. Participants provided standardized information and blood samples. Plasma and buffy coats were immediately prepared and frozen at -80°C until testing.

Plasma specimens were tested by using an ELISA for HCV antibodies to recombinant antigens c22, c200, and NS5B (ELISA-3.0, Ortho-Clinical Diagnostics, Raritan, NJ, USA) according to the manufacturer's instructions. To conserve samples we did not confirm positive results by RIBA but instead tested for virus in plasma by using a real-time reverse transcription (RT)–PCR assay for HCV RNA developed in our laboratory. This quantitative assay amplified a conserved 155-nucleotide target sequence within the HCV 5´ untranslated region and had an absolute sensitivity of 9 IU/mL of viral load (43 copies/mL). Total RNA was extracted from 140 μL plasma by using the Qiagen Viral RNA Mini kit (Qiagen, Valencia, CA, USA). RT-PCR was performed in a Thermo Hybaid MBS 0.2S (Fisher Scientific International, Hampton, NH, USA) in triplicate reactions that used 10 μL RNA per reaction. After cDNA synthesis, quantitative PCR was performed by using an ABI Prism 7700 or 7900 Sequence Detection System (Applied BioSystems, Foster City, CA, USA). HCV RNA–positive specimens were further characterized by sequencing parts of the Core/E1 and NS5B regions. Briefly, purified RNA was used to generate cDNA by reverse transcription. Nested PCR was performed with sets of published PCR primers to amplify DNA from Core/E1 or NS5B regions (*6**,**7*). The amplification products were separated in an agarose gel and purified by using the Promega DNA purification kit (Promega, Madison, WI, USA). The BigDye Terminator kit (Applied BioSystems) was used to prepare products for sequencing in an Applied BioSystems 310 automated sequencer.

**Figure 1:**
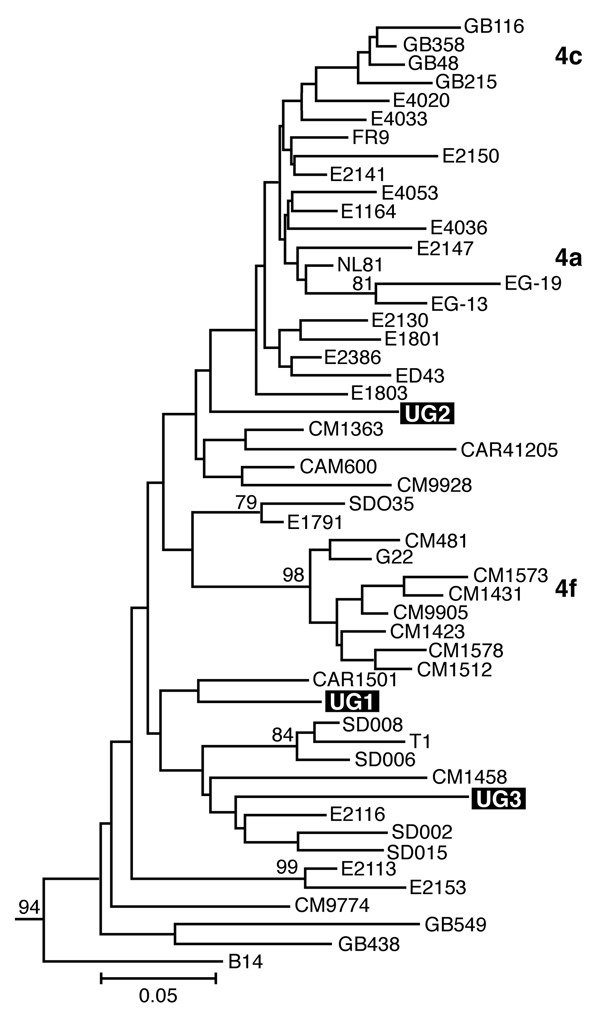
Estimated phylogenies of hepatitis C virus genotype 4; NS5B phylogenetic analysis based on 350 bp of NS5B nucleotide sequence. Ugandan sequences determined in this study are highlighted in black. Numerical values (presented when >60%) represent the statistical support for the tree topology as determined by 1,000 bootstrap replicates. Reference sequences for genotypes 1–3, 5, and 6 (*7*) were included in both analyses and retained as the outgroup. Accession numbers are provided in the text.

Separate alignments were generated for the NS5B and Core/E1 regions, each including sequences from our samples and genotype 1 to 6 reference sequences obtained from GenBank (*7*). Additional genotype 4 reference sequences used in the analysis are listed in Figure 1. These sequences were aligned in ClustalX (version 1.81, Plate-Forme de Bio-Informatique, Illkirch, France) by using the ClustalW (version 1.6, Plate-Forme de Bio-Informatique) matrix and edited in GeneDoc version 2.6 (http://www.psc.edu/biomed/genedoc). In Mega version 2.1 (http://www.megasoftware.net), the Clustal alignments for Core/E1 and NS5B were used to generate neighbor-joining trees by using the Kimura 2-parameter plus G distribution (K80+G) distance model. Free parameters were reduced to the K80 model, and α values were determined by using a maximum likelihood approach in PAUP*4.0 (Sinauer Associates, Inc. Publishers, Sunderland, MA, USA).

Of 603 children (median age 6.8 years, interquartile range 3.9–10.9 years), 27 (4%) were HCV antibody positive. No significant trends were found between HCV status and demographic (age, sex, tribe) or household (urban/rural location, water supply, room density) variables. Three (5%) of 61 children born to HCV-seropositive mothers were HCV seropositive, compared with 16 (3%) of 456 children born to seronegative mothers (p = 0.58). Eleven (3%) of 322 children with a history of transfusion (mean 1.5 transfusions, range 1–10) were HCV seropositive, compared with 12 (5%) of 245 children with no history of transfusion (p = 0.38). Prevalences in children with (2%) and without (4%) history of scarification were also similar (p = 0.38).

Using RT-PCR to test for HCV RNA, we examined 58 samples from children: all 27 samples with antibody-positive results, 11 with high-negative (near the positive cutoff) results, 10 with negative results from children with a history of frequent transfusions, and 10 with low-negative results from children with no history of transfusion. Only 3 samples, all from seropositive children (ages 6, 7, and 13 years), had positive results by RT-PCR, and viral RNA levels in these samples were 2.3×10^3^, 2.8×10^4^, and 3.8×10^6^ HCV IU/mL plasma.

Of 525 serum samples available from mothers, 62 (12%) were antibody positive. We sought HCV RNA in all 62 mothers with positive ELISA results and in 20 mothers with high-negative results. Five (8%) antibody-positive mothers (ages 28, 30, 38, 39, and 42 years) had RNA-positive samples. Viral levels varied from 6.6×10^3^ to 5.7×10^5^ HCV IU/mL (median 8.1×10^4^). All HCV-seronegative mothers (median age 32.3 years, mean 33.2 years, standard deviation 7.6) were RNA negative.

**Figure 2:**
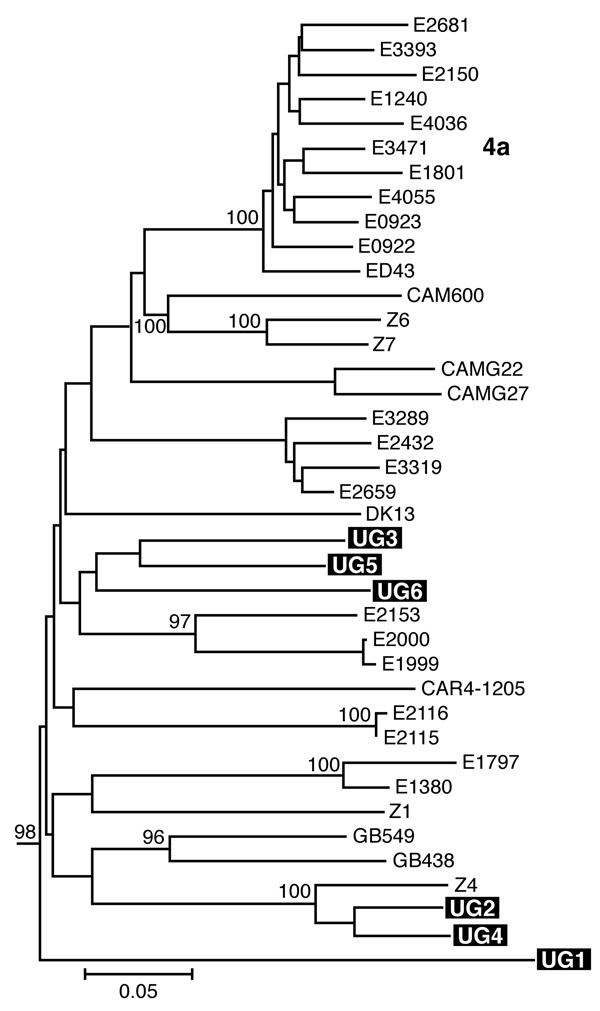
Estimated phylogenies of hepatitis C virus genotype 4; Core/E1 phylogenetic analysis based on 340 bp spanning the junction between the Core and E1 regions. Ugandan sequences determined in this study are highlighted in black. Numerical values (presented when >60%) represent the statistical support for the tree topology as determined by 1,000 bootstrap replicates. Reference sequences for genotypes 1–3, 5, and 6 (*7*) were included in both analyses and retained as the outgroup. Accession numbers are provided in the text.

We amplified samples for sequencing from 5 mothers and 1 child and obtained Core/E1 sequences for all 6 samples and NS5B sequences for 3 samples. None of the sequences was identical to any other or to any sequence in GenBank. Phylogenetic analysis of both the NS5B and Core/E1 regions showed that all viruses amplified clustered within genotype 4 (Figure 1 and Figure 2). In the Core/E1 phylogeny (Figure 2 ), 2 clusters of HCV strains and 1 outlier (UG1) appeared. Each of the NS5B sequences obtained (Figure 1) was from a different Core/E1 group, and they did not cluster. Sequences from this study have been deposited in GenBank (accession nos. AY577578–AY577586).

## Conclusions

We found HCV infection to be uncommon in Uganda, consistent with reports from other Ugandan studies. This cohort of patients with sickle cell disease had frequent blood transfusions, but only 4% of the children were HCV positive by ELISA. Of the seropositive children, only 11% had HCV RNA detectable by RT-PCR. Seropositivity in their mothers was higher, 12%, but only 8% of the seropositive women had positive RT-PCR results. If we assume that untested seronegative patients were not viremic, the overall prevalence of HCV RNA was 0.5% in children and 1% in mothers.

These results might indicate that false-positive ELISA results for HCV are frequent. False-positive results could be due to nonspecific antibody binding or to cross-reactivity with other tropical pathogens, e.g., flaviviruses found in Africa (*2**,**3*). Alternatively, perhaps true antibody-positive participants did not have positive RT-PCR results because they had cleared HCV viremia; however, in the industrialized world, most antibody-positive persons have persistent viremia. We did not confirm ELISA positivity with RIBA because, in Africa, few HCV ELISA-positive samples are confirmed by RIBA (*3**,**8*).

Our RT-PCR results confirm that HCV is present in Uganda, albeit at a low prevalence. How HCV is transmitted in sub-Saharan Africa is not known. We did not find correlations between infection in children and either seropositivity or viremia in their mothers. We expected to see iatrogenic transmission (*1**,**8*) because the children in this study all had sickle cell disease and more than half had had blood transfusions, and many had had multiple transfusions. Most children would have also had potential exposure to HCV from contaminated needles used during the course of their medical care or blades used in scarification by traditional healers (*9*). However, despite these potential exposures, documented HCV infections were uncommon.

Most HCV isolates can be classified into 6 major genotypes on the basis of sequence data from the NS5B, core, and E1 genomic regions. In this study, as in others (*8*), multiple sets of primers were needed to amplify these regions because different primer sets worked for different samples, which reflects the diversity of HCV. Our HCV Core/E1 tree showed 3 clusters. Neither the Core/E1 nor NS5B sequences in Uganda clustered closely with genotype 4 sequences reported from other areas (Figure 1 and Figure 2). Because the number of sequences was limited, we have not assigned subtypes to our newly described variants.

In sub-Saharan Africa, genotype frequencies vary by location (*10*). We found only genotype 4 in Uganda. Genotype 4 has been found in the west-central nations of Africa (Nigeria, Cameroon, Gabon, and Central African Republic), but it also occurs in Tanzania (*11*). Phylogenetic analysis shows that types 1 and 4 share a common ancestor (*12*). Ndjomou et al. (*13*) noted that genotypes 1 and 4 in Cameroon have considerable genetic diversity and suggested that these types may have arisen there. They postulate that genotype 4 spread east into central Africa. Our detection of HCV genotype 4 in Uganda is consistent with their hypothesis, but we do not know the direction of spread. Type 4 is also found in Egypt, where its epidemic distribution is attributed to widespread iatrogenic transmission during schistosomiasis eradication campaigns from 1960 to 1980 (in which needles for injecting potassium antimony tartrate were reused after rudimentary cleaning) (*7**,**14*), and in the Arabian Peninsula (*15*). For many centuries, Arab traders have had extensive contact with African populations of the Nile watershed region, including Uganda, which provides routes for genotype 4 to spread between East Africa and the Middle East.
